# Incidence of Immune-Mediated Pseudoprogression of Lymphoma Treated with Immune Checkpoint Inhibitors: Systematic Review and Meta-Analysis

**DOI:** 10.3390/jcm10112257

**Published:** 2021-05-23

**Authors:** Amy Junghyun Lee, Kyung Won Kim, Young Chul Cho, Yousun Ko, Yu Sub Sung, Youngbin Shin, Jiwoo Lee, Mi-hyun Kim

**Affiliations:** 1 Department of Medical Science, Asan Medical Institute of Convergence Science and Technology, Asan Medical Center, University of Ulsan College of Medicine, Seoul 05505, Korea; amyjung.lee89@gmail.com; 2 Department of Radiology, Asan Medical Center, University of Ulsan College of Medicine, Seoul 05505, Korea; dlwldncjs@gmail.com; 3 Biomedical Research Center, Asan Institute for Life Sciences, Asan Medical Center, Seoul 05505, Korea; cjsakura@naver.com (Y.C.C.); ko.yousun82@gmail.com (Y.K.); i.am.yongbin@gmail.com (Y.S.); 4 Department of Convergence Medicine, Clinical Research Center, Asan Medical Center, University of Ulsan College of Medicine, Seoul 05505, Korea; asmilez.sung@gmail.com; 5 Department of Radiation Science & Technology, Jeonbuk National University, Jeonju 54896, Korea; mhkimasan@gmail.com

**Keywords:** pseudoprogression, lymphoma, indeterminate response, Lugano, meta-analysis

## Abstract

We evaluated the incidence of pseudoprogression and indeterminate response (IR) in patients with lymphoma treated with immune checkpoint inhibitors (ICIs). A systematic search of PubMed and EMBASE was performed up to 6 February 2021, using the keywords “lymphoma,” “immunotherapy,” and “pseudoprogression.” Random-effects models were used to calculate both pooled incidence of pseudoprogression patients with lymphoma and an IR according to LYRIC criteria, while the Higgins inconsistency index (I2) test and Cochran’s Q test were used for heterogeneity. Eight original articles were included, in which the number of patients ranged from 7 to 243. Among the lymphoma patients with ICIs, the pooled incidence of pseudoprogression was 10% (95% confidence interval [CI]: 0.06–0.17). There was no publication bias in Begg’s test (*p* = 0.14). Three articles were analyzed to determine the pooled incidence of pseudoprogression in patients with IR according to LYRIC criteria in a subgroup analysis, which was shown to be 19% (95% CI: 0.08–0.40). A significant proportion (10%) of patients with lymphoma treated with ICIs showed pseudoprogression, and 19% of patients with an IR response showed pseudoprogression and a delayed response. Immune-related response criteria such as LYRIC may be used for patients with lymphoma treated with ICIs.

## 1. Introduction

Cancer immunotherapy using immune checkpoint inhibitors (ICIs) has dramatically changed the treatment of various malignancies, including solid tumors and lymphoma. Several ICIs have been approved that target cytotoxic T-lymphocyte-associated antigen 4 (CTLA-4) and programmed cell death protein 1 (PD-1) or its ligand (PD-L1) [1,2,3,4]. Extensive research over recent decades has revealed that pseudoprogression can occur in a subset of patients treated with ICIs. Pseudoprogression refers to the atypical tumor response pattern after an increase of tumor burden or the appearance of new lesions [5]. A recent meta-analysis demonstrated that the incidence of pseudoprogression in solid tumors was 6.0%, but its definition varies across studies and has not been standardized yet for solid tumors [6].

In the field of lymphoma treatment, the concept of pseudoprogression was adopted in the response criteria through a workshop conducted by the Lymphoma Research Foundation and the Cancer Research Institute. Thus, the LYmphoma Response to Immunomodulatory therapy Criteria (LYRIC) was proposed in 2016 [7], an updated version of the Lugano Classification Lymphoma Response Criteria [8]. The LYRIC used ‘indeterminate response (IR)’ to describe the initially increased tumor burden, which distinguished pseudoprogression from true progression on subsequent imaging or biopsy.

However, the incidence of pseudoprogression has not yet been well explored in patients treated with ICIs and among patients with an IR. Although there have been several individual studies, no attempt has yet been made to generate a systematic summary on the incidence of pseudoprogression in these patients. Therefore, we performed this systematic review and meta-analysis to determine the incidence of pseudoprogression in patients with lymphoma treated with ICIs.

## 2. Materials and Methods

This systematic review and meta-analysis adhered to the Preferred Reporting Items for Systematic Reviews and Meta-Analyses (PRISMA) guidelines [9].

### 2.1. Literature Search

A systematic search of the PubMed and EMBASE databases was conducted to identify relevant original articles up to 6 February 2021. The search keywords and their related terms were as follows: lymphoma AND (atypical AND response) OR (indeterminate response) OR pseudoprogression) AND (immune checkpoint OR immunotherapy OR PD-1 OR PD-L1 OR CTLA-4 OR CART-T). Bibliographies of retrieved articles were also screened, and there were no restrictions on studies conducted in any language.

Individual studies have used various types of terminologies to describe the pseudoprogression phenomenon, including an atypical response with a transient tumor increase, an IR not confirmed as progression, and pseudoprogression. In this meta-analysis, we grouped all of these terms under ‘pseudoprogression’.

### 2.2. Inclusion and Exclusion Criteria

Studies (or subsets of studies) that investigated the incidence of pseudoprogression in patients with lymphoma treated with ICIs were eligible for inclusion in the analysis. Studies (or subsets of studies) that satisfied the following criteria were included:

- Population: studies that included six or more patients with lymphoma treated with ICIs;
- Reference standard: studies that used tumor response criteria based on imaging;
- Study design: clinical trials and observational studies (i.e., retrospective or prospective);
- Outcomes: results were reported in sufficient detail to evaluate the incidence of pseudoprogression among patients treated with ICIs.

The exclusion criteria included the following:

- Case reports and series with sample sizes of less than five patients and studies;
- Review articles, editorials, letters, comments, and conference proceedings;
- Studies with topics that deviated from the incidence of pseudoprogression in lymphoma;
- Studies with insufficient data for evaluating the incidence of pseudoprogression.

### 2.3. Quality Assessment

The risk of bias and methodological quality was evaluated using the Risk Of Bias In Nonrandomized Studies of Interventions (ROBINS-I) [10] for nonrandomized studies, including observational studies.

### 2.4. Data Extraction

The data from the included studies were extracted as follows: demographic and clinical information of patients (patient numbers, mean age, sex, drug name, and type of lymphoma), study information (authors, title, study periods, and study type), and outcome information (number of patients with pseudoprogression, patients with LYRIC IR, and outcome of pseudoprogression).

### 2.5. Statistical Analysis

The pooled incidence of pseudoprogression among patients with lymphoma treated with ICIs was obtained using a random-effects model and an inverse-variance weighting model [11]. Heterogeneity was evaluated using the Higgins inconsistency index (I^2^) test and Cochran’s Q test [12,13,14,15]. I^2^ values greater than 50% indicated substantial heterogeneity [13]. Publication bias was assessed using a funnel plot and Begg’s test. All reported P-values are 2-sided, with findings of *p* < 0.05 considered to be statistically significant. The robustness of the results was tested with a sensitivity analysis performed by recalculating the pooled estimates after excluding each study.

Subgroup analyses were performed for the pooled incidence of pseudoprogression among patients with an IR according to LYRIC and treated with ICIs only, excluding the CAR-T. All statistical analyses were performed using the “meta” packages in R version 3.6.3 (R Foundation for Statistical Computing, Vienna, Austria) [11].

## 3. Results

### 3.1. Literature Search

**Figure 1:**
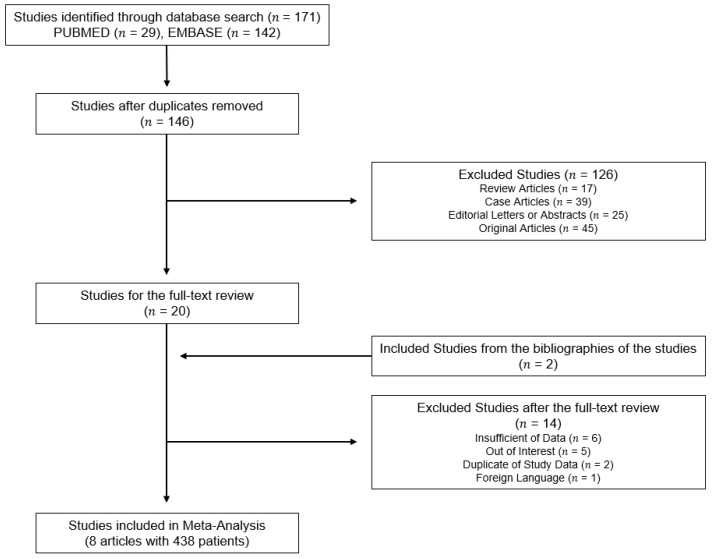
Flow Diagram of the Study Selection.

Our literature search process is described in Figure 1. The eligibility articles from PubMed and the EMBASE database totaled 146 articles after removing duplicates. After a review of the titles and abstracts, 127 of 146 articles were excluded: 17 review articles, 39 case articles, 25 editorials or abstracts, and 45 original articles that did not align with the purpose of our study. Of these, 20 full-text articles were retrieved. After a full-text review, 14 articles were further excluded: six articles with insufficient data for pseudoprogression, five articles for the out-of-study topic, two articles with overlapping study data, and one article in a foreign language. There were two additional articles through the bibliography search of these articles. Finally, eight articles [16,17,18,19,20,21,22,23] were included for the qualitative and quantitative analysis.

### 3.2. Study Characteristics and Quality Assessment

Table 1 represents the study characteristics of the final eight articles with a total of 438 patients. There were six retrospective studies and two clinical trials. The number of patients with relapsed or refractory Hodgkin lymphoma, non-Hodgkin lymphoma, and NK/T cell lymphoma across all studies ranged from 7 to 243. In most studies (*n* = 7), patients were treated with single ICIs such as nivolumab, pembrolizumab, or CAR-T cell therapy. In contrast, patients in Merryman’s study received a single ICI or a combination of ICIs (e.g., nivolumab and ipilimumab, or other PD-(L1) combinations). Half of the studies used Lugano 2014 as response criteria [16,17,18,19], and the remaining half used the five-point Deauville score [20], immune Response Evaluation Criteria in Solid Tumours (iRECIST) [21], the revised response criteria [22], or International Working Group (IWG) group [23]. Among the eight included studies, three used Lugano and LYRIC criteria together [16,17,18].

**Figure 2:**
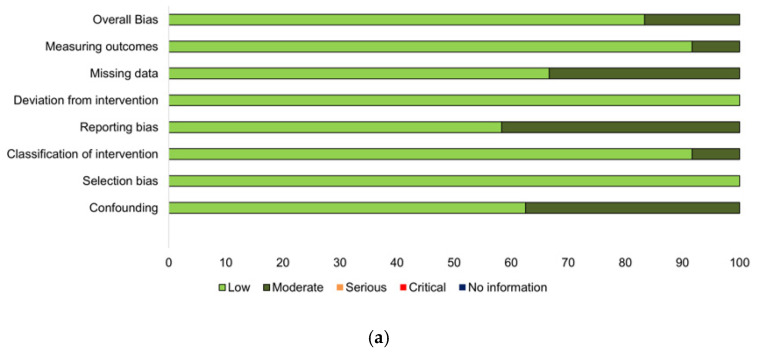

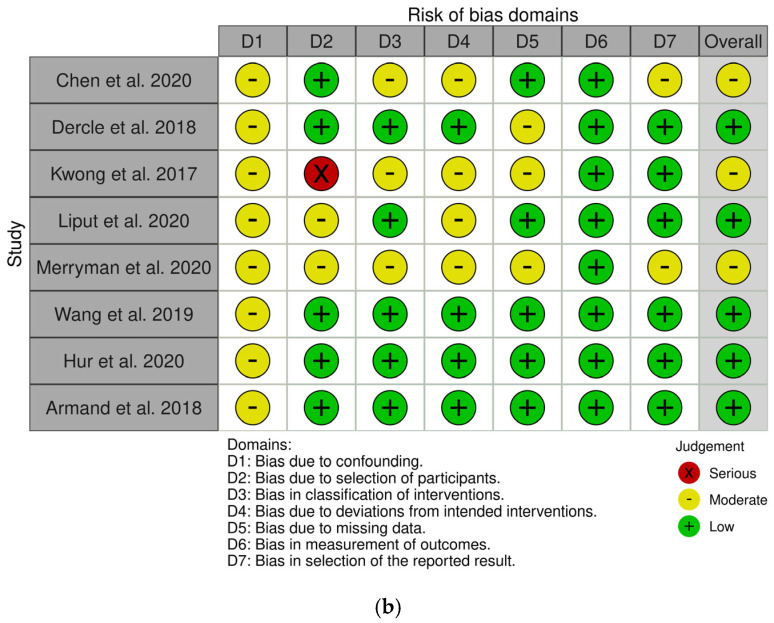
Risk of Bias in Nonrandomized Studies of Intervention (ROBINS-I) for Quality Assessment. (**a**) Distribution of risk of bias results within each bias domain; (**b**) description of the domain level results for each study.

According to the result of the ROBINS-I as a nonrandomized quality assessment, there were five low-risk studies and three moderate-risk studies (Figure 2).

### 3.3. Meta-Analysis

**Figure 3:**
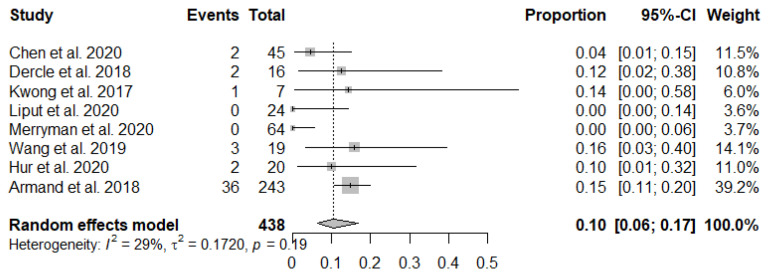
Forest plots to show the pooled incidence of pseudoprogression.

**Figure 4:**
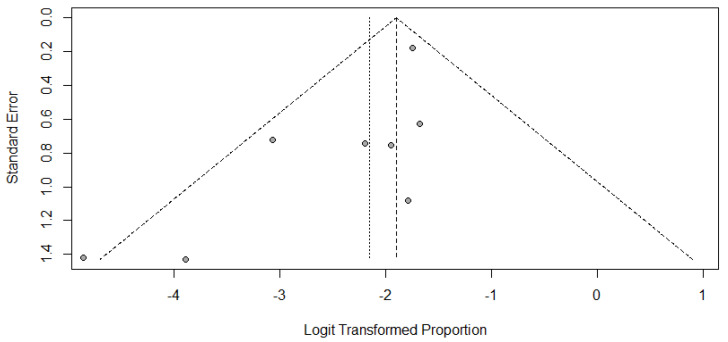
Funnel plots for visual appraisal of the literature bias.

The forest plots of incidence of pseudoprogression are presented in Figure 3. The pooled incidence of pseudoprogression among patients with lymphoma treated with ICIs was 10.4% (95% confidence interval [CI]: 6.2–16.8%) (Figure 3). The sensitivity analysis revealed the robustness of recalculated pooled incidence of pseudoprogression after excluding each study ranging from 8.3% to 13.4%. There was no publication bias in the funnel plot (Figure 4) and Begg’s test (*p* = 0.14).

**Figure 5:**
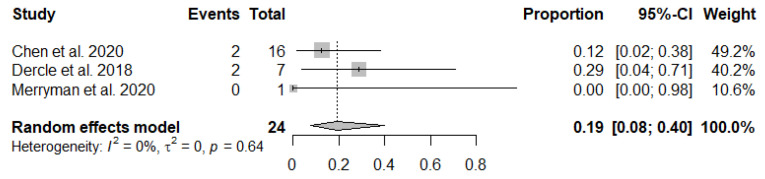
Forest plots show the pooled incidence of pseudoprogression among patients with an indeterminate response.

**Figure 6:**
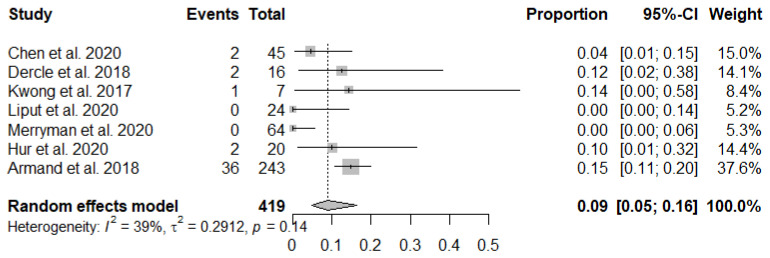
Forest plots show the pooled incidence of pseudoprogression among patients treated with only ICIs.

The first subgroup analysis revealed that three studies evaluated the IR according to LYRIC. The pooled incidence of pseudoprogression among patients with an IR was 19.1% (95% CI: 7.7–40.1%) (Figure 5). Seven studies using only ICIs were analyzed for the second subgroup analysis, which showed that the pooled incidence rate of pseudoprogression was 9.0% (95% CI: 4.7–16.3%) (Figure 6) [16,17,18,20,21,22,23].

## 4. Discussion

Our systematic review demonstrated that the incidence of pseudoprogression was 10.4% (95% CI: 6.2–16.8%) among all patients receiving ICIs for lymphoma. The subgroup analysis showed that 19.1% (95% CI: 7.7–40.1%) showed pseudoprogression among patients with an IR according to lyric LYRIC.

The 10.4% incidence of pseudoprogression in patients with lymphoma treated with ICIs was somewhat higher than that of patients with solid tumors. A recent meta-analysis reported that the incidence of pseudoprogression in solid tumors was 6.0% (95% CI: 5.0–7.0) [6], which may be attributable to the characteristics of lymphoid malignancies and immunotherapy. Indeed, it has been reported that the immunomodulatory agents used before the era of ICIs, particularly lenalidomide, may cause “tumor flare” in a large proportion of patients (up to 15%) with chronic lymphocytic leukemia small lymphocytic lymphoma, which may be related with an immune reaction between immune cells such as natural killer cells and malignant lymphoid cells [7]. However, the exact mechanism of pseudoprogression in lymphoma treated with ICIs has not been well explored.

Our study results demonstrate that 19.1% of patients with IR showed pseudoprogression and delayed response. Our study favors adopting the LYRIC in addition to the Lugano criteria for tumor response assessment in patients with lymphoma treated with ICIs. The Lugano criteria have been widely used for response assessment of lymphoma. However, the concept of pseudoprogression is not reflected in the Lugano criteria. Thus, the LYRIC criteria have been issued to reflect pseudoprogression in lymphoma patients treated with immunotherapy by using the IR category. However, the LYRIC criteria have suffered from a lack of validation due to insufficient evidence. Our meta-analysis results can provide systematic evidence for the incidence of pseudoprogression among all lymphoma patients as well as among patients with IR category.

The strict application of the Lugano criteria alone could result in the incorrect assignment of progressive disease (PD), leading to early cessation of therapy before ICI clinical benefit can be achieved. The LYRIC criteria proposed the IR category, which allows ICI treatment to be continued in patients with PD according to the Lugano criteria, so-called “treatment beyond progression.” The LYRIC mandated a subsequent imaging assessment for patients with IR within 12 weeks to confirm or refute PD [7]. So far, LYRIC has not commonly been used in clinical practice and clinical trials for lymphoma treated with ICIs. Indeed, only three out of eight studies have adopted LYRIC for their tumor response assessment in our systematic review. We hope that our results can support the adoption of LYRIC in patients with lymphoma treated with ICIs.

The potential drawbacks of LYRIC include the complexity of the rules and the relatively long interval (12 weeks) between initial and subsequent imaging assessment. There are three categories in IR, i.e., IR(1), IR(2), and IR(3), with complex definitions [3,7]. Even in our imaging core lab (Asan Image Metrics, www.aim-aicro.com), dedicated central readers and image analysts for lymphoma response criteria had difficulty following the LYRIC guidelines. Medical oncologists usually do not want to spend much time following LYRIC. We hope that a more simplified version of LYRIC will be developed to increase the accessibility of the pseudoprogression concept to healthcare providers for patient management, gain insights into the pseudoprogression phenomenon, and prevent early cessation of ICIs for potential responders.

There are several limitations to this study. First, we used a small sample size. However, our study is, to the best of our knowledge, the first systematic review based on all available literature at the time of writing and is timely. Second, most included studies were retrospective in nature, warranting further large-scale studies or additional accumulated evidence. Third, it was difficult to perform proper subgroup analysis for the pseudoprogression rate according to lymphoma subtypes due to insufficient data from included literature.

## 5. Conclusions

In conclusion, a significant proportion (10.4%) of patients with lymphoma treated with ICIs showed pseudoprogression, and almost all included studies have presented evidence to support the pseudoprogression phenomenon. Our systematic review results strongly favor using immune-related response criteria such as LYRIC for patients with lymphoma treated with ICIs.

## Figures and Tables

**Table 1:** Characteristics of Studies Included in the Meta-Analysis.

Author, Year of Publication	Location	Study Design	Response Criteria	Category of Pseudoprogression	Type of Lymphoma	Agent	No. of Patients	Patients with Pseudoprogression	Patients with IR
Chen et al. 2020 [16]	France	Retrospective	Lugano, LYRIC	Progressive disease followed by transient progression in lesions size and metabolism on LYRIC	Relapsed or Refractory HL	Nivolumab	45	2	16
Dercle et al. 2018 [17]	France	Clinical Trial	Lugano, LYRIC	Progressive disease followed by transient progression on LYRIC	Relapsed or Refractory HL	Pembrolizumab or Nivolumab	16	2	7
Kwong et al. 2017 [20]	Hong Kong, Singapore, South Korea	Retrospective	5-point Deauville Score	Progressive Disease followed by CR on 5-point Deauville Score	Relapsed or Refractory NK/T-cell Lymphoma	Pembrolizumab	7	1	N/A
Liput et al. 2020 [21]	USA	Retrospective	iRECIST	N/A	Relapsed or Refractory Classical HL and Non-HL	Pembrolizumab or Nivolumab	24	0	N/A
Merryman et al. 2020 [18]	USA	Retrospective	Lugano, LYRIC	N/A	HL	ICIs (Pembrolizumab, Nivolumab, Ipilimumab, Combination, or other PD-[L]1)	64	0	1
Wang et al. 2019 [19]	China	Retrospective	PERCIST, Lugano	Progressive disease followed by PR or CR on PERCIST	Relapsed or Refractory Non-HL	CAR-T cell Therapy	19	3	N/A
Armand et al. 2018 [22]	Europe, North America	Multicenter, Phase II Clinical Trial	IWG Criteria	treated beyond initial progression followed by PR or CR on IWG Criteria	Relapsed or Refractory Classical HL	Nivolumab	243	36	N/A
Hur et al. 2020 [23]	South Korea	Retrospective	The revised response criteria	Progressive disease followed by CR on the revised response criteria	Classical HL	Pembrolizumab or Nivolumab	20	2	N/A

* IR, Indeterminate Response; LYRIC, the LYmphoma Response to Immunomodulatory therapy Criteria; HL, Hodgkin Lymphoma; CR, Complete Response; iRECIST, immune Response Evaluation Criteria in Solid Tumours; PR, Partial Response; ICIs, Immune Checkpoint Inhibitors; PD-1, Programmed cell Death protein 1; PD-L1, Programmed Death Ligand 1; PERCIST, Positron Emission Tomography (PET) Response Criteria in Solid Tumors; CAR-T, Chimeric Antigen Receptor T cell; IWG, International Working Group; N/A, Not Applicable.

## Data Availability

The data presented in this study are openly available in databases: PUBMED and EMBASE.

## References

[1] Galligan B.M., Tsao-Wei D., Groshen S., Kirschabum M., O’Donnell R., Kaesberg P.R., Siddiqui T., Popplewell L., Sikander A., Myo H. (2015). Efficacy and safety of combined rituximab and ipilimumab to treat patients with relapsed/refractory CD20+ B-cell lymphoma. Blood.

[2] Lang D., Wahl G., Poier N., Graf S., Kiesl D., Lamprecht B., Gabriel M. (2020). Impact of PET/CT for Assessing Response to Immunotherapy-A Clinical Perspective. J. Clin. Med..

[3] Lepik K.V., Mikhailova N.B., Moiseev I.S., Kondakova E.V., Tsvetkova L.A., Zalyalov Y.R., Borzenkova E.S., Babenko E.V., Baykov V.V., Markova I.V. (2019). Nivolumab for the treatment of relapsed and refractory classical Hodgkin lymphoma after ASCT and in ASCT-naive patients. Leuk Lymphoma.

[4] Lunning M.A., Moskowitz A.J., Horwitz S. (2013). Strategies for relapsed peripheral T-cell lymphoma: The tail that wags the curve. J. Clin. Oncol..

[5] Seymour L., Bogaerts J., Perrone A., Ford R., Schwartz L.H., Mandrekar S., Lin N.U., Litière S., Dancey J., Chen A. (2017). iRECIST: Guidelines for response criteria for use in trials testing immunotherapeutics. Lancet Oncol..

[6] Park H.J., Kim K.W., Pyo J., Suh C.H., Yoon S., Hatabu H., Nishino M. (2020). Incidence of Pseudoprogression during Immune Checkpoint Inhibitor Therapy for Solid Tumors: A Systematic Review and Meta-Analysis. Radiology.

[7] Cheson B.D., Ansell S., Schwartz L., Gordon L.I., Advani R., Jacene H.A., Hoos A., Barrington S.F., Armand P. (2016). Refinement of the Lugano Classification lymphoma response criteria in the era of immunomodulatory therapy. Blood.

[8] Cheson B.D., Fisher R.I., Barrington S.F., Cavalli F., Schwartz L.H., Zucca E., Lister T.A. (2014). Recommendations for initial evaluation, staging, and response assessment of Hodgkin and non-Hodgkin lymphoma: The Lugano classification. J. Clin. Oncol..

[9] Moher D., Liberati A., Tetzlaff J., Altman D.G., Group P. (2010). Preferred reporting items for systematic reviews and meta-analyses: The PRISMA statement. Int. J. Surg..

[10] Sterne J.A., Hernán M.A., Reeves B.C., Savović J., Berkman N.D., Viswanathan M., Henry D., Altman D.G., Ansari M.T., Boutron I. (2016). ROBINS-I: A tool for assessing risk of bias in non-randomised studies of interventions. BMJ.

[11] Viechtbauer W. (2010). Conducting meta-analyses in R with the metafor package. J. Stat. Softw..

[12] Higgins J.P.T., Altman D.G., Gøtzsche P.C., Jüni P., Moher D., Oxman A.D., Savović J., Schulz K.F., Weeks L., Sterne J.A.C. (2011). The Cochrane Collaboration’s tool for assessing risk of bias in randomised trials. BMJ.

[13] Higgins J.P.T., Thompson S.G., Deeks J.J., Altman D.G. (2003). Measuring inconsistency in meta-analyses. BMJ.

[14] Kim K.W., Lee J., Choi S.H., Huh J., Park S.H. (2015). Systematic Review and Meta-Analysis of Studies Evaluating Diagnostic Test Accuracy: A Practical Review for Clinical Researchers-Part I. General Guidance and Tips. Korean J. Radiol..

[15] Lee J., Kim K.W., Choi S.H., Huh J., Park S.H. (2015). Systematic Review and Meta-Analysis of Studies Evaluating Diagnostic Test Accuracy: A Practical Review for Clinical Researchers-Part II. Statistical Methods of Meta-Analysis. Korean J. Radiol..

[16] Chen A., Mokrane F.Z., Schwartz L.H., Morschhauser F., Stamatoullas A., Schiano de Colella J.M., Vercellino L., Casasnovas O., Chauchet A., Delmer A. (2020). Early 18F-FDG PET/CT Response Predicts Survival in Relapsed or Refractory Hodgkin Lymphoma Treated with Nivolumab. J. Nucl. Med.: Off. Publ. Soc. Nucl. Med..

[17] Dercle L., Seban R.D., Lazarovici J., Schwartz L.H., Houot R., Ammari S., Danu A., Edeline V., Marabelle A., Ribrag V. (2018). (18)F-FDG PET and CT Scans Detect New Imaging Patterns of Response and Progression in Patients with Hodgkin Lymphoma Treated by Anti-Programmed Death 1 Immune Checkpoint Inhibitor. J. Nucl Med..

[18] Merryman R.W., Carreau N.A., Advani R.H., Spinner M.A., Herrera A.F., Chen R., Tomassetti S., Ramchandren R., Hamid M., Assouline S. (2020). Impact of Treatment Beyond Progression with Immune Checkpoint Blockade in Hodgkin Lymphoma. Oncologist.

[19] Wang J., Hu Y., Yang S., Wei G., Zhao X., Wu W., Zhang Y., Zhang Y., Chen D., Wu Z. (2019). Role of Fluorodeoxyglucose Positron Emission Tomography/Computed Tomography in Predicting the Adverse Effects of Chimeric Antigen Receptor T Cell Therapy in Patients with Non-Hodgkin Lymphoma. Biol. Blood Marrow Transplant..

[20] Kwong Y.L., Chan T.S.Y., Tan D., Kim S.J., Poon L.M., Mow B., Khong P.L., Loong F., Au-Yeung R., Iqbal J. (2017). PD1 blockade with pembrolizumab is highly effective in relapsed or refractory NK/T-cell lymphoma failing L-asparaginase. Blood.

[21] Liput J., Guler E., Smith D.A., Tirumani S.H., Hoimes C., Caimi P.F., Ramaiya N.H. (2020). Clinical, Imaging Findings, Responses, and Outcomes of Patients With Classical Hodgkin Lymphoma and Non-Hodgkin Lymphoma Undergoing Immune Checkpoint Inhibitor Therapy: A Single-Institution Experience. J. Comput. Assist. Tomogr..

[22] Armand P., Engert A., Younes A., Fanale M., Santoro A., Zinzani P.L., Ansell S.M. (2018). Nivolumab for Relapsed/Refractory Classic Hodgkin Lymphoma After Failure of Autologous Hematopoietic Cell Transplantation: Extended Follow-Up of the Multicohort Single-Arm Phase II CheckMate 205 Trial. J. Clin. Oncol..

[23] Hur J.Y., Yoon S.E., Kim S.J., Kim W.S. (2020). Immune checkpoint inhibitors in patients with pretreated HodgkinEs lymphoma: A Korean single-center, retrospective study. Blood Res..

